# Robotic-Assisted Thoracoscopic Repair of Chronic Bronchoesophageal Fistula Using Omental Flap Interposition: A Case Report

**DOI:** 10.7759/cureus.77973

**Published:** 2025-01-25

**Authors:** Emily M Su, Justin Smith, Logan T Mellert, Maureen E Cheung

**Affiliations:** 1 General Surgery, Summa Health, Akron, USA; 2 Bariatric and Minimally Invasive Surgery, Summa Health, Akron, USA; 3 Cardiothoracic Surgery, Summa Health, Akron, USA

**Keywords:** bronchoesophageal fistula, minimally invasive surgery, omental flap, robotic-assisted thoracoscopic surgery, thoracoscopy

## Abstract

Bronchoesophageal fistulas (BEFs) are rare and challenging conditions caused by congenital or acquired factors, often requiring innovative surgical solutions. This report describes the successful management of a chronic, postsurgical BEF in a 44-year-old male using a combination of robotic-assisted thoracoscopic surgery and laparoscopic omental flap interposition. The patient’s complex history of failed esophageal stenting and dense pleural adhesions highlighted the need for a minimally invasive yet precise approach. Preoperative optimization, intraoperative technical considerations, and the use of the da Vinci Xi robotic system facilitated successful fistula closure and omental flap placement. Postoperative recovery was uneventful, with no recurrence at follow-up. This case highlights the value of robotic-assisted thoracoscopy and omental flap interposition in managing challenging BEFs, offering a promising alternative to traditional thoracic muscle flaps. This novel application demonstrates the potential to enhance surgical precision, minimize morbidity, and improve patient outcomes for complex fistulas.

## Introduction

Bronchoesophageal fistulas (BEFs) represent abnormal connections between the bronchial tree and the esophagus. These rare but serious conditions may arise from congenital or acquired causes. Congenital BEFs are typically associated with esophageal atresia and manifest immediately postnatally, while acquired BEFs are more common in adults and result from malignancy, infection, trauma, or iatrogenic causes, including surgery and radiation therapy [1,2]. Effective management of BEFs depends on their etiology and patient condition, with treatment options ranging from endoscopic stenting to innovative surgical techniques such as robotic-assisted thoracoscopic surgery [3-9].

This report details a case of a BEF likely resulting from a combination of pleural adhesions and altered anatomy from previous thoracic surgeries compounded by chronic inflammation from recurrent pneumonia and tobacco use. This BEF was repaired using robotic-assisted thoracoscopy and laparoscopic omental flap interposition - a novel approach that demonstrates favorable outcomes. The decision-making process, technical considerations, and postoperative results are discussed to provide a comprehensive understanding of this technique.

## Case presentation

**Figure 1:**
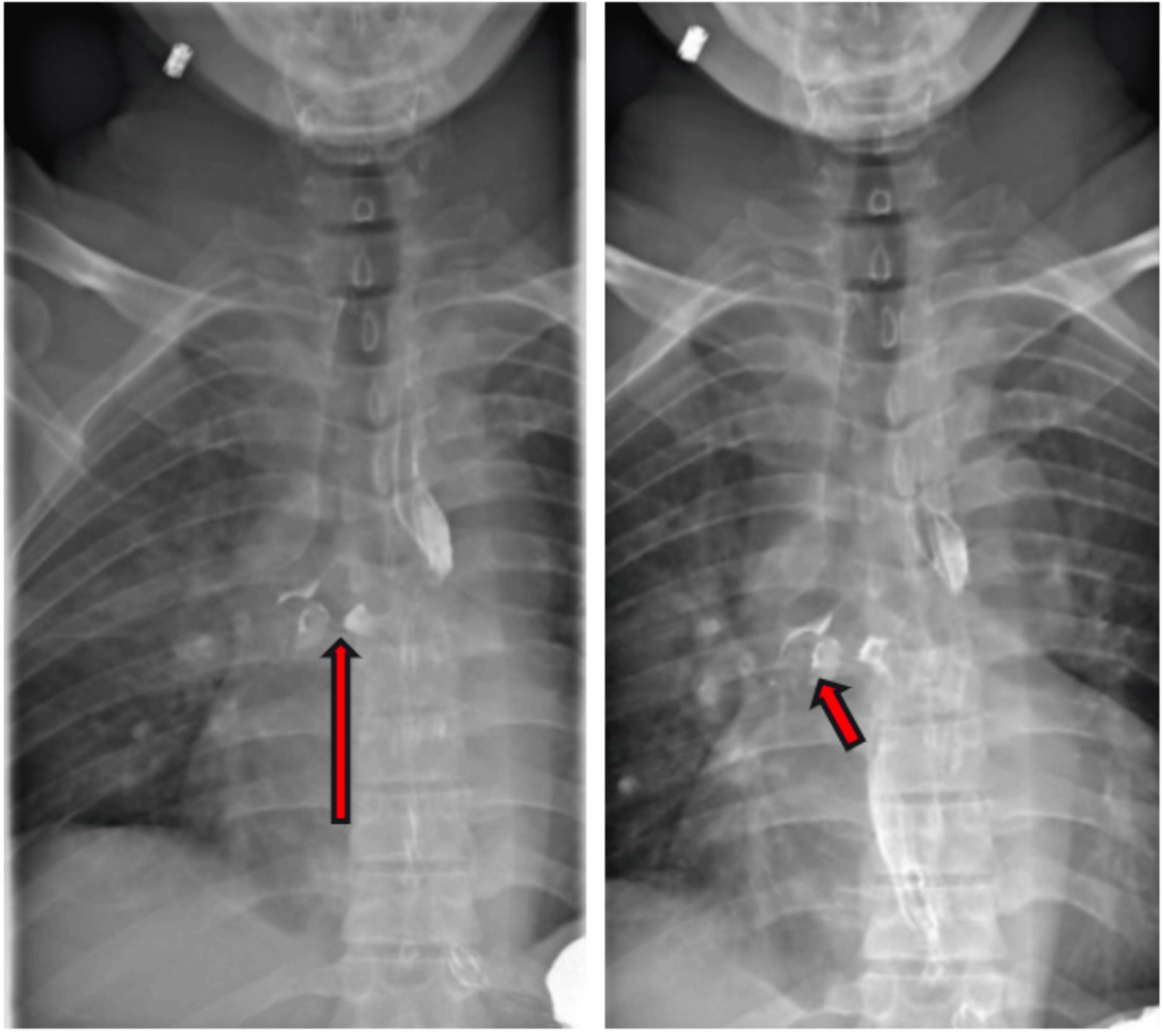
(Left, right) Barium esophagram showing a small diverticulum extending off the mid esophagus and irregularity of the diverticulum with contrast extension into the right mainstem bronchus suggesting BEF (red arrows). BEF: Bronchoesophageal fistula

**Figure 2:**
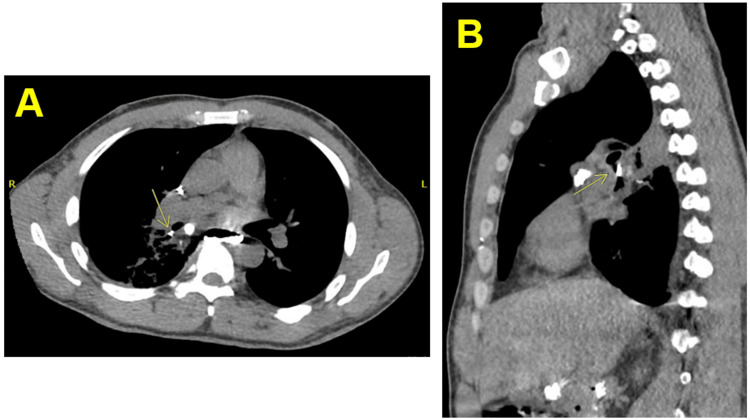
Axial (A) and sagittal (B) CT of the abdomen and pelvis demonstrating oral contrast in the right mainstem and adjacent right lower lobe bronchus compatible with BEF (yellow arrows), right hilar fullness with bronchial narrowing and wall thickening, and increased opacity along the posterior medial right lower lobe with pleural thickening. BEF: Bronchoesophageal fistula

**Figure 3:**
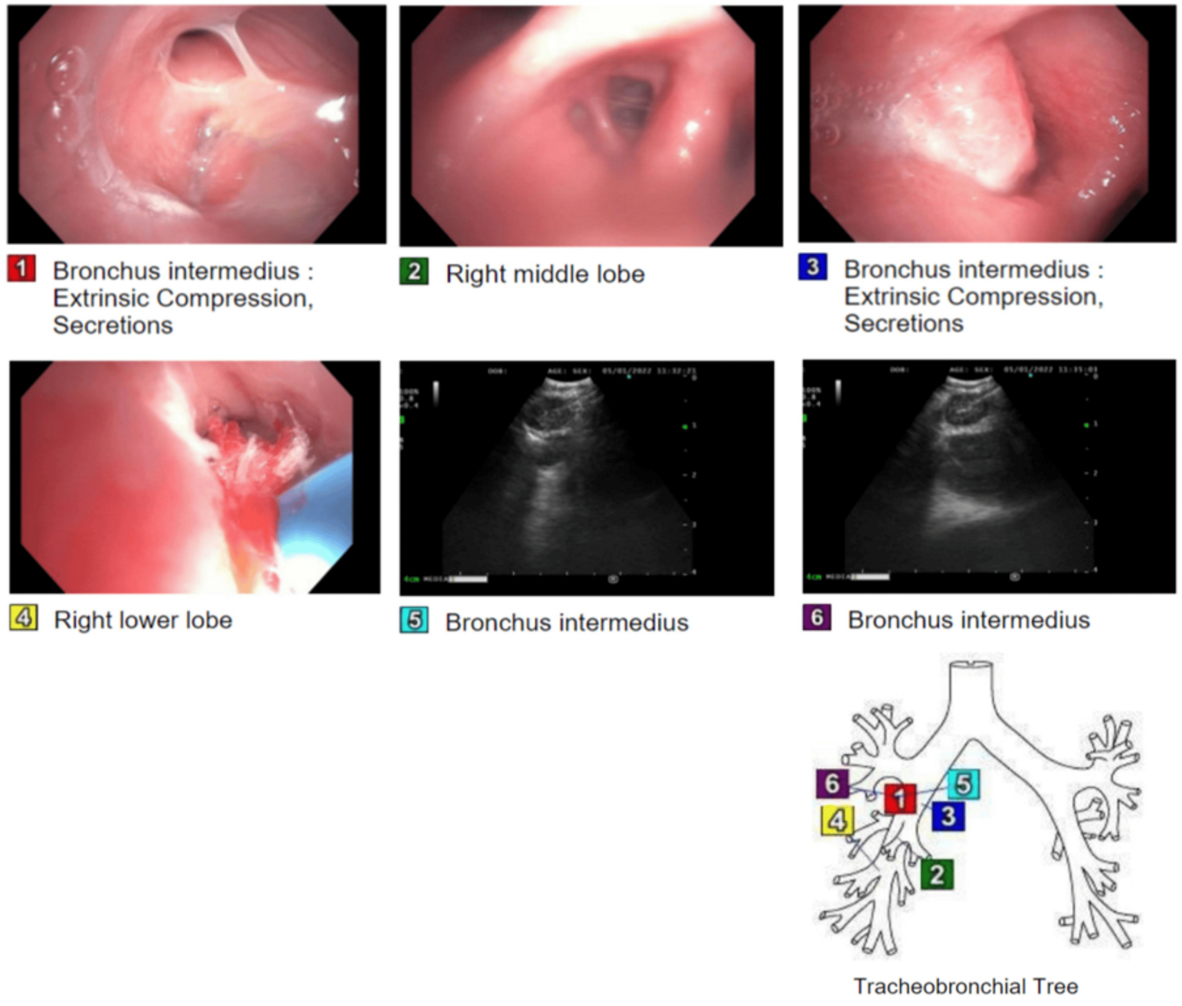
Bronchoscopy identifying a right hilar mass, extrinsic compression of the bronchus intermedius with the airway lumen nearly occluded secondary to posterior mass effect, and notable secretions throughout the right tracheobronchial tree. Transbronchial needle aspiration, lung biopsy, brushings, bronchoalveolar lavage, and endobronchial ultrasound performed. This image is the original work of the authors.

A 44-year-old male with a history of tobacco use and emphysema presented with chronic cough, reflux, and recurrent right lower lobe pneumonia, symptoms consistent with a BEF. Diagnostic studies, including esophagram, computed tomography (CT), and bronchoscopy, confirmed a fistulous connection between the mid-esophagus and right mainstem bronchus (Figures 1-3). Initial management involved three esophageal stent placements, all failing due to migration and discomfort. The patient subsequently opted for surgical intervention.

Rationale for a minimally invasive approach

Given the patient’s history of pleural adhesions and prior pleurectomy, a robotic-assisted thoracoscopic approach was chosen for its ability to provide superior visualization and precision in challenging anatomical areas [10]. Strategic preoperative planning and intraoperative techniques mitigated concerns regarding port placement and insufflation in the context of dense adhesions. Adhesions were encountered during initial port placement, particularly at the prior thoracotomy site, and were carefully managed with blunt dissection and meticulous use of electrocautery to create a working space.

The decision to use an omental flap over alternatives such as intercostal or latissimus dorsi muscle flaps was based on several considerations. The omental flap’s rich vascularity and immunologic properties make it an ideal choice for promoting healing and reducing the risk of infection [11]. Additionally, its versatility and ease of mobilization with laparoscopic techniques offer a less invasive alternative to thoracic muscle harvesting [8,12-14]. While the omental flap may complicate future esophagectomy, this risk was discussed with the patient preoperatively, and the benefits of using a well-vascularized tissue were deemed to outweigh the potential drawbacks [5].

Operative technique

Preoperative preparation included 11 days of total parenteral nutrition to optimize the patient’s nutritional status. The surgical plan involved a laparoscopic omental flap harvest followed by robotic-assisted thoracoscopic repair.

During the laparoscopic phase, the patient was positioned supine. The left upper quadrant was accessed at Palmer’s point with an optical trocar, and three additional 5 mm trocars were placed under direct visualization in the epigastrium, left lateral abdomen, and right upper quadrant (RUQ). The omentum was manipulated cephalad over the liver to expose and free its attachments to the transverse colon. Then, the omentum was manipulated caudad and mobilized off of the greater curvature of the stomach proximal to the right gastroepiploic artery, which was carefully preserved as the pedicle blood supply for the omental flap. Extreme care was taken to avoid excess traction and any splenic injury. The left gastroepiploic artery was divided. Upon inspection of the RUQ, the gallbladder appeared to have chronic changes and moderate adhesions; therefore, a cholecystectomy was performed, and the final pathology confirmed chronic cholecystitis. The flap was prepared for transposition into the thoracic cavity, and pneumoperitoneum was evacuated at the end of this phase.

**Figure 4:**
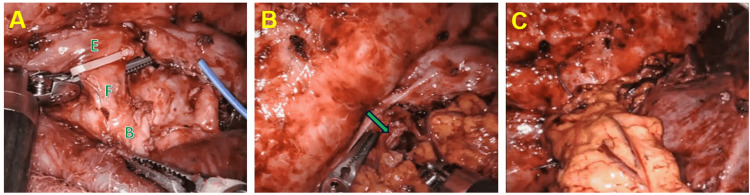
(A) Isolation of fistula (F) between bronchus (B) and esophagus (E) with clip near esophageal orifice. (B) After BEF division, omental flap being positioned (clip can be visualized at green arrow tip). (C) Omental flap interposition. BEF: Bronchoesophageal fistula

For the thoracoscopic phase, the patient was repositioned in the left lateral decubitus position, and the right lung was isolated. An 8-mm thoracoscopic incision in the eighth intercostal space at the posterior axillary line was made, and the subcutaneous and adipose tissue were divided to enter the pleural space under direct visualization. An 8-mm robotic port was placed through the incision, and the camera was introduced. After insufflation, two additional 8 mm robotic ports were placed in the fifth and seventh intercostal spaces, and a 12 mm air seal assistant port was placed in the ninth intercostal space. The da Vinci Xi robot was docked; the camera, long bipolar, and cautery instruments were introduced; and the mediastinal dissection and fistula identification were begun. Dense adhesions from prior surgeries were encountered in the mediastinum, particularly near the bronchus intermedius. These were carefully dissected to expose and isolate the fistulous tract (Figures 4A-4C). Flexible bronchoscopy and esophagoscopy were used intraoperatively to confirm the fistula’s location and extent.

The fistulous tract was circumferentially dissected, but we were unable to safely traverse the tract with a robotic stapler. Therefore, we elected to place a clip on the BEF near the esophageal orifice to prevent mucosal retraction, and the fistula was divided and sutured closed along the tract itself with 3-0 Vicryl suture and at both esophageal and bronchial orifices, with 3-0 Prolene suture at the latter. The esophageal muscular layer was closed with a 3-0 Stratafix PDS suture. The omental flap was then tacked to the posterior mediastinum at the level of the right mainstem bronchus with a 2-0 Ethibond suture for adequate coverage of the fistula area and interposition between the esophagus and bronchus intermedius. Injection of intravenous indocyanine green demonstrated good flap perfusion and appropriate pedicle orientation without torsion or tension. A leak test of the bronchial repair was negative. A 24FR straight chest tube was directed toward the apex, and a 24FR Blake drain was positioned along the diaphragm and posterior mediastinum near the repair site.

The patient experienced an uneventful postoperative course. His straight chest tube was removed postoperative day 3 once it was confirmed to have no air leak on the water seal and output less than 30 cc per day. He underwent an upper gastrointestinal esophagram postoperative day 4 that showed no leak, and the Blake chest tube was removed postoperative day 6 after it also had no air leak on the water seal and output less than 30 cc per day. He was transitioned to a soft diet postoperative day 6 prior to discharge after a 17-day hospitalization. The patient underwent a barium esophagram 15 months later while he was briefly admitted for endemic coronavirus, which showed no evidence of BEF recurrence.

## Discussion

The management of BEFs remains a clinical challenge due to their complexity and potential for significant morbidity. The etiology of BEFs is a crucial factor in determining the appropriate treatment approach. Acquired BEFs, such as the one in this case, can arise from a variety of causes, including malignancy, infection, trauma, or iatrogenic injury from prior surgeries or radiation therapy [1,2]. In this patient, the BEF was likely the result of a combination of postsurgical factors, including pleural adhesions and altered anatomy from previous thoracic surgeries, compounded by chronic inflammation from recurrent pneumonia and tobacco use. Chronic inflammation is particularly significant as it promotes tissue friability and disrupts normal healing processes, predisposing to the formation of abnormal fistulous tracts. The presence of dense pleural adhesions and altered mediastinal anatomy in this patient not only contributed to the formation of the BEF but also posed significant challenges during surgical repair. These factors highlight the importance of preoperative planning and the selection of a minimally invasive robotic-assisted approach to navigate complex anatomical distortions safely. The choice of this approach underscores the utility of robotic systems in managing difficult anatomical scenarios, particularly in patients with extensive prior thoracic surgical histories.

The clinical significance of BEFs extends beyond their immediate symptoms, such as recurrent aspiration pneumonia, which was a prominent feature in this patient’s presentation. Left untreated, BEFs can lead to severe complications, including sepsis, malnutrition, and progressive respiratory failure, all of which can significantly impair quality of life and increase mortality risk. This underscores the critical need for timely and effective interventions to prevent these life-threatening sequelae.

Endoscopic stenting is often considered a first-line option for benign or small fistulas; but in this case, the failure of three separate esophageal stent placements due to migration and discomfort highlighted the limitations of this modality [3,4,6]. These failures emphasized the need for a more definitive and durable surgical solution.

Robotic-assisted thoracoscopy provided an ideal platform for addressing this challenging case. Its enhanced visualization and precision allowed for meticulous dissection of the fistulous tract and management of dense adhesions while minimizing collateral tissue damage. The use of indocyanine green fluorescence imaging to confirm omental flap perfusion exemplifies the integration of advanced technologies to optimize surgical outcomes. By ensuring adequate blood supply to the interposed flap, this technique reduces the risk of fistula recurrence and enhances the likelihood of successful healing.

The selection of an omental flap was particularly significant in this case. The omental tissue’s rich vascularity and immunologic properties made it an excellent choice for interposing between the esophagus and bronchus, providing a robust barrier to prevent recurrence [11]. While muscle flaps are more commonly used, the patient’s prior thoracic surgeries and extensive adhesions made the omental flap a more feasible and less invasive option [12-14]. This decision not only reduced operative morbidity but also avoided the additional risks associated with muscle flap harvesting.

**Figure 5:**
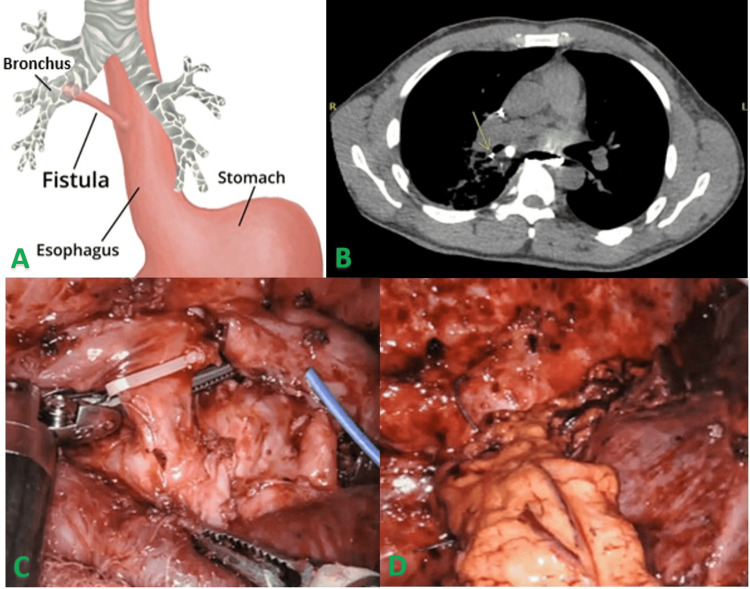
Summary of bronchoesophageal fistula (BEF, A) requiring laparoscopic omental flap harvest and right robotic-assisted thoracoscopy with omental flap interposition. Oral contrast CT showing contrast in the bronchial tree (B). Intraoperative images showing isolation and clipping of the BEF prior to division (C) and then omental flap interposition over the area (D). Key technical pearls for successful execution included meticulous flap mobilization to preserve vascular integrity, careful handling during delivery into the thoracic cavity, and ensuring appropriate pedicle orientation without torsion. These steps are critical to maintaining perfusion and optimizing healing of the fistula repair. This image is the original work of the authors.

Finally, the success of this novel robotic-assisted thoracoscopic approach with omental flap interposition in achieving durable fistula closure highlights its potential as a valuable option for managing complex or recurrent BEFs. By combining the advantages of minimally invasive techniques with the biological benefits of omental tissue, this approach offers a promising avenue for improving patient outcomes and advancing the surgical management of this challenging condition (Figures 5A-5D).

## Conclusions

Robotic-assisted thoracoscopy with omental flap interposition is a novel and effective approach to BEF repair. By combining the advantages of minimally invasive robotic surgery with the biological benefits of omental tissue, this technique offers a promising alternative for addressing complex or recurrent fistulas. The favorable outcomes achieved in this case highlight its potential to improve patient recovery and reduce complications, advancing the surgical management of this challenging condition.
